# Identification of Minimal Pairs of Japanese Pitch Accent in Noise-Vocoded Speech

**DOI:** 10.3389/fpsyg.2022.887761

**Published:** 2022-05-31

**Authors:** Yukiko Sugiyama

**Affiliations:** Faculty of Science and Technology, Keio University, Yokohama, Japan

**Keywords:** Japanese, lexical pitch accent, speech perception, secondary cues, noise-vocoded speech

## Abstract

The perception of lexical pitch accent in Japanese was assessed using noise-excited vocoder speech, which contained no fundamental frequency (*f*_*o*_) or its harmonics. While prosodic information such as in lexical stress in English and lexical tone in Mandarin Chinese is known to be encoded in multiple acoustic dimensions, such multidimensionality is less understood for lexical pitch accent in Japanese. In the present study, listeners were tested under four different conditions to investigate the contribution of non-*f*_*o*_ properties to the perception of Japanese pitch accent: noise-vocoded speech stimuli consisting of 10 3-ERBN-wide bands and 15 2-ERBN-wide bands created from a male and female speaker. Results found listeners were able to identify minimal pairs of final-accented and unaccented words at a rate better than chance in all conditions, indicating the presence of secondary cues to Japanese pitch accent. Subsequent analyses were conducted to investigate if the listeners' ability to distinguish minimal pairs was correlated with duration, intensity or formant information. The results found no strong or consistent correlation, suggesting the possibility that listeners used different cues depending on the information available in the stimuli. Furthermore, the comparison of the current results with equivalent studies in English and Mandarin Chinese suggest that, although lexical prosodic information exists in multiple acoustic dimensions in Japanese, the primary cue is more salient than in other languages.

## 1. Introduction

Early studies in English found that not only segmental but also prosodic information is encoded across multiple acoustic dimensions (Cooper et al., 1952; Delattre et al., 1955; Fry, 1955, 1958; Harris et al., 1958). For example, a voicing contrast between voiced and voiceless stop consonants is primarily realized by the difference in voice onset time (VOT) word-initially, but it is also present in other acoustic dimensions, such as fundamental frequency (*f*_*o*_), the frequency of the first formant, and the intensity of burst at onset (Liberman et al., 1958; Lisker and Abramson, 1964; Kingston and Diehl, 1994). Since these acoustic properties systematically co-vary with the voicing contrast, listeners can identify the intended phoneme even when one cue is compromised, using the remaining cues (Best et al., 1981; Pisoni and Luce, 1987).

At a suprasegmental level, classic studies by Fry (1955) and Fry (1958) show that duration, intensity and *f*_*o*_ all contribute to the perception of lexical stress in English. English has a number of words whose parts of speech are distinguished by the location of lexical stress. For example, when the phoneme sequence /ɑbʤɛkt/ has lexical stress on its first syllable, it is a noun. By contrast, it is a verb when it has lexical stress on the second syllable. When a syllable is stressed, it tends to have a longer duration, a greater intensity, and a greater change in *f*_*o*_, in addition to change in vowel quality (Lehiste, 1970). When one of the cues is neutralized or absent, listeners can use the remaining cues to identify the words they heard. Research in other Indo-European languages including languages such as Dutch (Sluijter et al., 1997), German (Kohler, 2012), Polish (Jassem, 1959), Russian (Chrabaszcz et al., 2014), and Spanish (Ortega-Llebaria and Prieto, 2011), has also found that lexical stress is characterized by multiple acoustic correlates (see Gordon and Roettger, 2017 for a recent cross-linguistic review).

Furthermore, the past few decades have seen a growing body of research in Mandarin Chinese showing that lexical tone is also realized through multiple acoustic properties (Blicher et al., 1990; Whalen and Xu, 1992; Xu et al., 2002; Kong and Zeng, 2006) While *f*_*o*_ is the dominant cue (Fu and Zeng, 2000), duration and amplitude contour contribute to the perception of lexical tone (Whalen and Xu, 1992; Xu et al., 2002; Kong and Zeng, 2006). Fu et al. (1998) created noise-vocoded speech using the algorithm described in Shannon et al. (1995) and found that the tone recognition accuracy was over 60 percent even when only one bandpass filter was used to extract temporal envelope information from the original speech. The accuracy reached over 65 percent with the four-band condition. Whalen and Xu (1992) used signal-correlated noise stimuli to investigate the contribution of amplitude contour and duration to word identification when words consisted of the same phoneme sequence and could be distinguished solely by tone. Although accuracy differed to some extent depending on the tone type, listeners were able to identify tone reasonably well using either the duration or amplitude information remaining in the stimuli. There are also studies that report a correlation between tones and phonation types (Gordon and Ladefoged, 2001; Garellek and Keating, 2011; DiCanio, 2012; Kuang, 2013; Brunelle and Kirby, 2016). Gordon and Ladefoged (2001) observe that, while creaky voice occurs with either a high tone or a low tone, breathiness occurs more consistently with lowered tones. Kuang (2013) conducted production and perception experiments in Black Miao, a language with five level tones which belongs to the Hmong-Mien family, and showed that some tones differ not only in *f*_*o*_ but also in terms of breathiness and creakiness, and listeners use these acoustic properties to distinguish lexical tones. Readers may notice that tone languages discussed in this kind of research are mainly so called contour-tone languages. In addition to tone, some researchers consider that Mandarin Chinese has stress (Duanmu, 2007). Therefore, studies that examined Mandarin Chinese may also include the effects of stress, but their possible effects are not clearly discussed in these studies. Languages spoken in Africa are also tonal but have a different type of tone known as register tone (Hyman, 2009). So far, research of this kind seems to be limited to contour-tone languages.

All these studies mentioned above show that lexical information is typically encoded across multiple acoustic dimensions, whether it is segmental or prosodic in nature. In Tokyo Japanese, lexical pitch accent is realized as a steep fall in *f*_*o*_ after accented syllables. Unlike English in which lexical stress is obligatory, words that have no accent (unaccented words) are possible in Japanese (Hyman, 2006). However, lexical prominence in both languages are similar in that words can have at most one lexical accent in Japanese and words can have at most one lexical stress in English. Furthermore, the location of lexical accent or lexical stress is used to distinguish words. Lexical pitch accent in Tokyo Japanese and lexical tone in Mandarin Chinese differ in that the former requires at least two syllables to realize lexical contrasts whereas monosyllabic words distinguished solely by their tones are possible in the latter. Unlike lexical pitch accent in Japanese, every syllable in a word must be specified for its tone in Mandarin Chinese. However, lexical pitch accent and lexical tone are similar in that they mainly manifest themselves in *f*_*o*_ (Cutler et al., 1997). Lexical prosody of Japanese is interesting in that it shares some common aspects with languages which are considered to have distinct lexical prosody both phonetically and functionally, such as stress in English and tone in Mandarin Chinese. For this reason, Tokyo Japanese has been analyzed accentually and tonally (e.g., McCawley, 1968; Haraguchi, 1977; Pierrehumbert and Beckman, 1988; Kawahara, 2015). Compared to stress in Indo-European languages and tone in Mandarin Chinese, much less is known about the acoustic details and perceptual cues of lexical pitch accent in Japanese. Findings in other languages are helpful in uncovering acoustic details of lexical pitch accent in Japanese.

As mentioned in the preceding paragraph, the primary cue to lexical pitch accent in Tokyo Japanese is *f*_*o*_ (Beckman, 1986; Kubozono, 1993), but other cues have not been clearly identified. Previous studies have looked at properties such as amplitude (Weitzman, 1970; Beckman, 1986; Cutler and Otake, 1999) and duration (Beckman, 1986; Kaiki et al., 1992; Cutler and Otake, 1999) as possible correlates of pitch accent. These studies show that amplitude might be somewhat correlated with pitch accent, but duration in general does not seem to be correlated with pitch accent. Beckman (1986) and Cutler and Otake (1999) recorded three types of disyllabic words: 1) words that had accent on their first syllable, 2) words that had accent on their second syllable and 3) words that had no accent, but found little correlation between accent and duration. While Itahashi (2005) notes that vowel duration is longer in accented syllables than in unaccented ones, the details of the relevant research are not indicated in his description. On the other hand, Kaiki et al. (1992) analyzed 503 sentences produced by four Japanese speakers (a total of 2,012 sentences) and concluded that accent had little influence on vowel duration.

For amplitude, Beckman (1986) recorded six minimal pairs of disyllabic words that contrast between initial accent and final accent. When peak amplitude and mean amplitude between the first syllables and second syllables were calculated, no consistent difference was found between the two types of words. Since it was not clear from production studies whether secondary cues to pitch accent exist in Japanese, Sugiyama (2017) conducted a perception study to determine if they actually exist. Using a Linear Predictive Coding (LPC) technique, Sugiyama separated the source information (*f*_*o*_ and its harmonics) existing in naturally produced speech from the filter information (formants). Then, the filter information obtained was excited by white noise to create the stimuli. The stimuli created this way were correctly identified at a rate that exceeded chance level with a mean *d'* score of 0.83 by native Japanese listeners, suggesting the presence of secondary cues.

The present study has three goals. First, using a different technique from the one employed in Sugiyama (2017), it aims to replicate the results found in Sugiyama (2017) to show that its results were not an artifact of the way the stimuli were created and secondary cues to Japanese pitch accent exist. Sugiyama (2017) created stimuli that contained no *f*_*o*_ or its harmonics using an LPC technique, the method adopted in Liu and Samuel (2004). In the present study, a sub-band coding technique described in Shannon et al. (1995) was used to create noise-excited vocoder speech. In creating the stimuli, the original speech signal was first filtered into multiple frequency bands, after which the amplitude envelope extracted from each band was used to modulate white noise. Noise-vocoded speech created this way preserves temporal and amplitude envelope information while degrading spectral information, removing *f*_*o*_ and its harmonics. If listeners can identify minimal pairs that contrast only in the presence or absence of accent, it would indicate that, like prosodic properties such as stress and tone, Japanese pitch accent is also realized through multiple acoustic correlates.

Second, the present study examines how the degree of degradation affects pitch perception by dividing speech signals into different numbers of filters. Because the LPC-based excitation method used in the previous study does not involve decomposition of spectral information into multiple channels, compared to the method used in the present study, it is possible that it provides more precise envelope energy information over the spectrum. More precisely speaking, temporal modulation in the energy envelope may have transmitted some harmonic information in the highest parts of the envelope modulation frequencies. By contrast, noise-based channel-vocoded speech is expected to provide a better control in removing pitch and harmonic information, as each envelope is low-pass filtered in terms of its modulation frequency components. Since *f*_*o*_ information is lexical in Tokyo Japanese, it is expected that removing *f*_*o*_ and its harmonics would severely affect word identification, at least compared to stress languages such as English. In the present study, noise-vocoded speech was created by dividing natural speech into 10 frequency bands with each band being 3-ERBN wide (equivalent rectangular bandwidths; Glasberg and Moore, 1990) and 15 frequency bands with each band being 2-ERBN bandwidths to investigate how word identification would be affected by different degrees of spectral degradation.

Third, using both male and female voices, the effect of speaker sex on the intelligibility of degraded speech was investigated. Males and females typically differ in their *f*_*o*_ with males having a lower *f*_*o*_ than females (Peterson and Barney, 1952). To the extent that the primary cue to Japanese lexical pitch accent is *f*_*o*_ and its harmonics, one might wonder why speaker gender should matter since they had been removed in the stimuli used in the current study. However, it is worth investigating because Loizou et al. (1998) found that vowels produced by men were easier to identify than those produced by women and children for cochlear implant users. Considering that noise-vocoded speech simulates hearing of cochlear implant users, male voice and female voice may have different effects on listeners' word identification in noise-vocoded speech. This is plausible also in light of the fact that male and female larynges are different. Because of the physiological differences, males and females differ in vocal characteristics such as how frequently creaky voice and breathy voice occur in their normal speech (e.g. Titze, 1989; Klatt and Klatt, 1990). These differences might lead to different intelligibility in noise-vocoded speech. While the acoustic difference between male and female voices resulting from physiological differences suggests that male voices may be more intelligible than female voices, Marguiles (1979) found that female voices were more intelligible than male voices in various noise conditions. The reasons for this finding are not stated clearly, but given that female speakers have a tendency toward more careful articulation (Beinum, 1980), some sociolinguistic factor may also play a role in speech intelligibility.

The findings from the present study will contribute to revealing acoustic characteristics of lexical pitch accent in Japanese. From the perspective of realizing robust speech communication, it is natural to expect that lexical pitch accent has some acoustic redundancy, just like lexical tone and lexical stress are realized through multiple acoustic properties. Furthermore, the present study contributes to understanding to what extent the acoustic properties that characterize human speech can be universal across languages irrespective of their prosodic types and, at the same time, to what extent they may be language-specific.

## 2. Experiment 1: Perception Study

### 2.1. Materials

The test words were five minimal pairs of final-accented words and unaccented words which have the same phoneme sequence within a pair (Table 1). Phonologically, final-accented words and unaccented words both have the pitch pattern of low-high and differ only in the presence or absence of accent on their final syllables. The most notable difference between the two types of words would appear in the following particle, when there is one. The particle has a low pitch when it follows an accented word while it has a high pitch when it follows an unaccented word. The five pairs of words were chosen from a larger set of words used in Sugiyama (2012), in which bimoraic test words were selected based on familiarity ratings provided in the NTT database (Lexical properties of Japanese, Amano and Kondo, 1999), so that all words have high familiarity ratings (5.0 or higher on a 7.0-scale, in which 7.0 indicates the highest familiarity). Because pitch accent is a lexical property and not predictable in Japanese, it was important that the words to be tested were familiar to the participants. The original set of words in Sugiyama (2012) contained nouns derived from verbs and nouns that typically occur in compounds as long as their familiarity ratings were 5.0 or higher. In conducting experiments, care was taken to ensure that carrier sentences would be compatible with any type of word as much as possible. Even so, participants in previous studies had difficulty interpreting the carrier sentences when nouns derived from verbs and nouns that typically occur in compounds occurred as test words. While it was desirable to include as many words as possible as test materials, it would be difficult to interpret the results when the listeners' judgements were influenced by the acceptability of the sentence. Therefore, these types of words were excluded in the present study. All test words were produced in the carrier sentence, *watashi wa* — *ga suki*, I TOPIC — NOM like, “I like —.” This carrier phrase was chosen because test words would be followed by a nominative case, which would be optimal for examining the effect of accent in the preceding test words, and it can be semantically compatible with a variety of words.

**Table 1:** Test words used in the experiment.

	**Final-accented**	**Orthography**	**Meaning**	**Unaccented**	**Orthography**	**Meaning**
1.	/han´a /	花	flower	/hana/	鼻	nose
2.	/has´i /	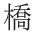	bridge	/hasi/	はし端	edge
3.	/hat´i /	八	eight	/hati/	蜂	bee
4.	/os´u/	おす雄	male	/osu/	お酢	vinegar
5.	/sit´a /	舌	tongue	/sita/	下	bottom

*Note: The only difference between final-accented words and unaccented words within each pair is the presence or absence of accent in the word-final syllable. The Chinese characters (kanji) 端 and 雄 were presented with the superscripts in hiragana to indicate how they should be read because other ways of reading them are also common*.

### 2.2. Stimuli

**Figure 1:**
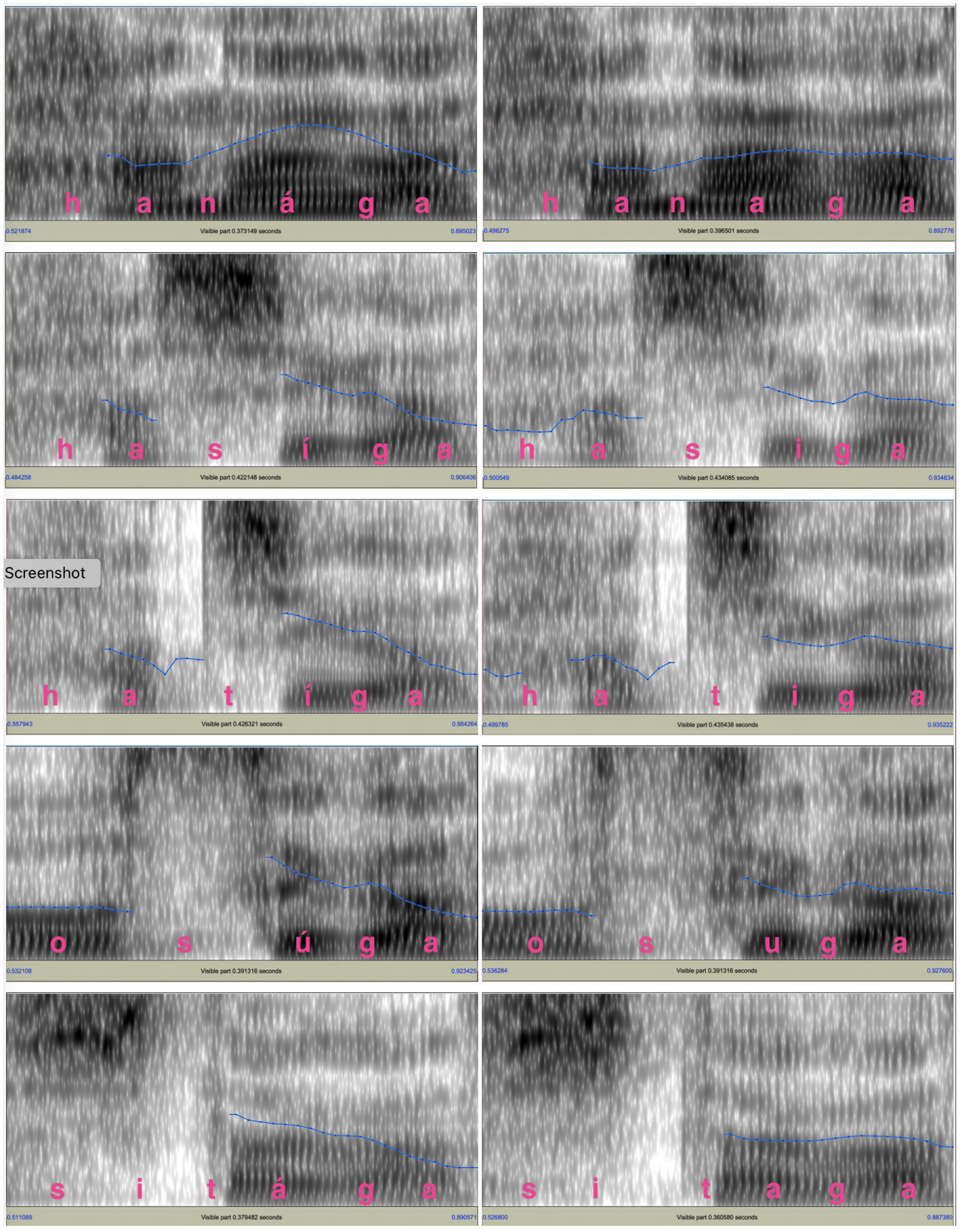
*f*_*o*_ contours of the test words and the following particle for the male voice. The blue lines in the spectrograms indicate the *f*_*o*_ movements. The *f*_*o*_ range is between 75 and 200 Hz.

**Figure 2:**
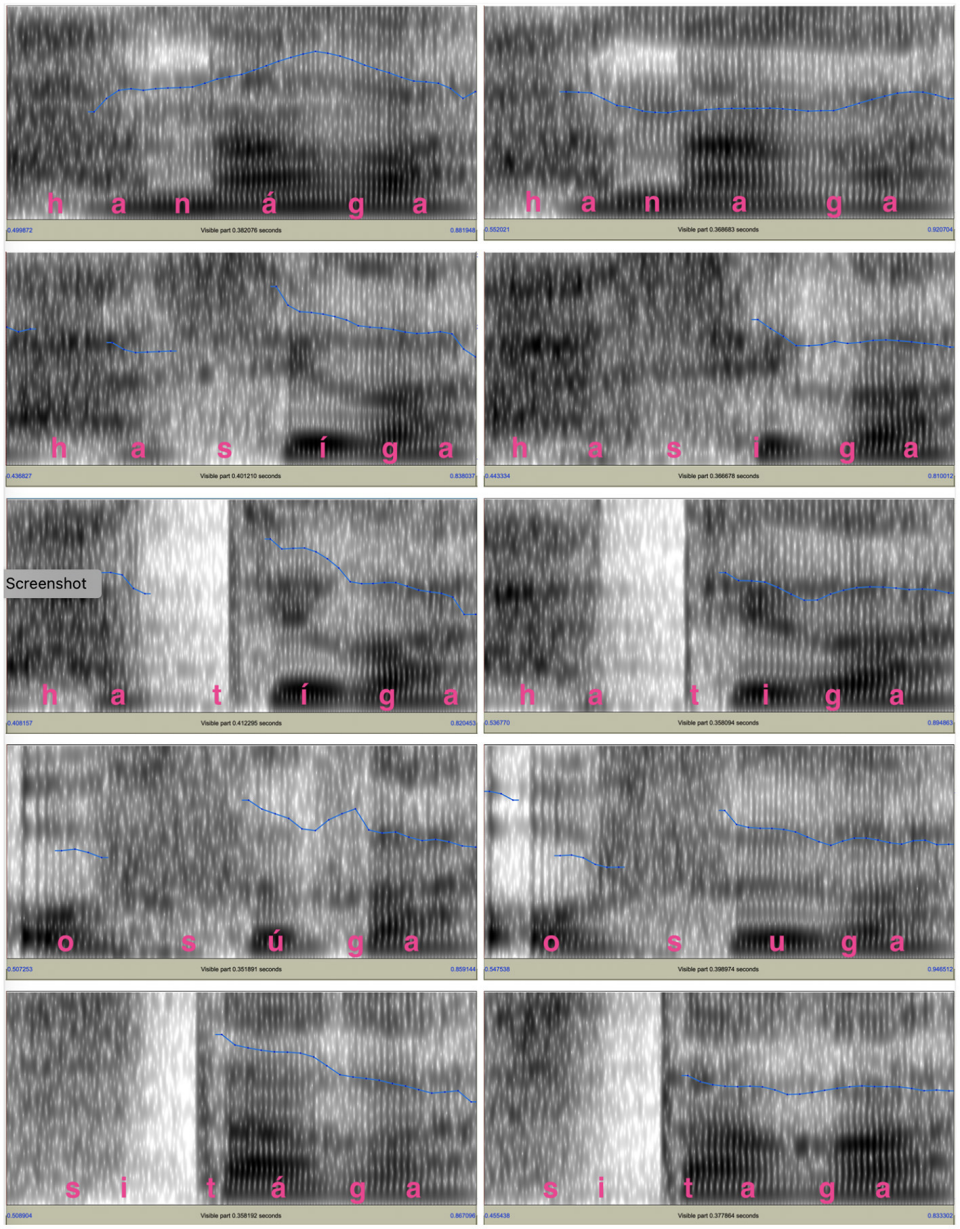
The *f*_*o*_ contours of the test words and the following particle for the female voice. The blue lines shown in the spectrograms indicate the *f*_*o*_ movements. The *f*_*o*_ range is between 75 Hz and 300 Hz for the *f*_*o*_s and 0 to 5,000 Hz for the spectrograms.

One male speaker and one female speaker recorded the test words in the carrier sentence. They were native speakers of Tokyo Japanese whose parents also grew up in the area where Tokyo Japanese was spoken. Both of them were college students in their early twenties with no professional training in speaking. Figures 1, 2 show the spectrograms and *f*_*o*_ contours for the test words and the following particle produced by the two speakers. For both figures, the panels on the left side show the *f*_*o*_ contours for final-accented words and the panels on the right side show the *f*_*o*_ contours for unaccented words. For the final-accented words, the *f*_*o*_ reaches its peak around the accented syllable and falls into the following particle. For the unaccented words, the *f*_*o*_ stays relatively flat between the word-final syllable and the following particle and does not have a clear peak.

**Figure 3:**
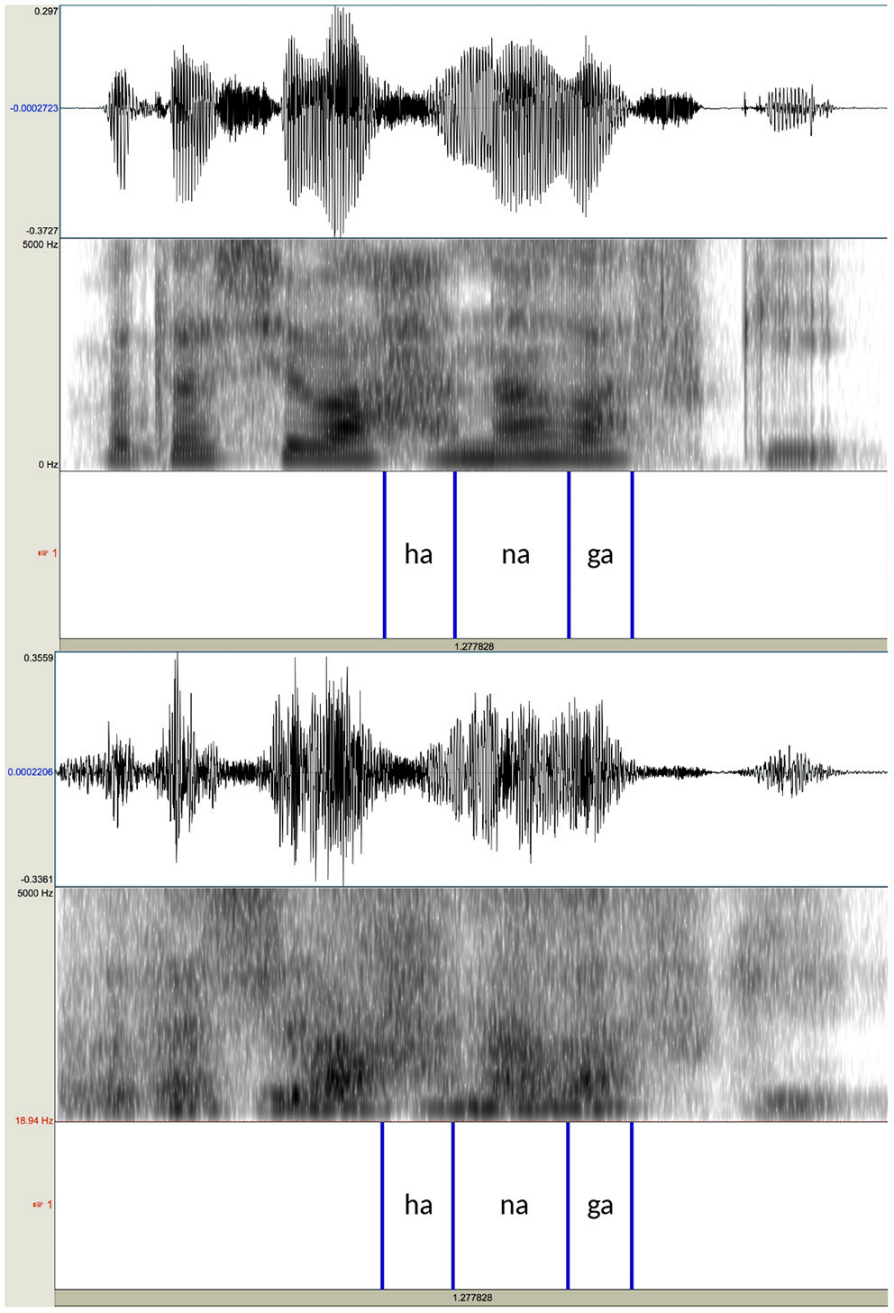
Waveforms and spectrograms of the sentence /watasi wa haná ɡa sukí/ ‘I prefer flowers' produced by the female speaker (upper panel) and its noise-vocoded version with the bandwidth of 2 ERBN (lower panel). The annotations at the bottom of each panel indicate the locations of the test words and their following particles.

The utterances were digitally recorded into a computer at 44.1 kHz with 16 bit resolution through an AKG C 4500 B-BC microphone, which was connected to a Mac Book Pro through an Apogee Duet audio interface. After recording, the sentences produced by the two speakers were noise-vocoded in Praat (Boersma and Weenink, 2019). In creating the noise-vocoded speech, modified versions of a script originally written by Darwin (2005) were used. Two kinds of stimuli, 10-band stimuli and 15-band stimuli, were created for both male and female voices. First, the recorded sentences were band-pass filtered into either 10 or 15 contiguous equivalent rectangular bandwidths (ERBN; Glasberg and Moore, 1990). The filters were divided in such a way so that the filtering would simulate the frequency selectivity of normal hearing individuals. In terms of ERBN, each frequency band had the bandwidth equivalent to 3 ERBN wide for the 10-band stimuli and 2 ERBN wide for the 15-band stimuli. The exact cutoff frequency values are shown in Table 2. Speech modified this way preserves much of the envelope information, which can be seen as slowly changing overall shape of waveforms, while removing pitch information, the rapidly changing temporal fine structure (Rosen, 1992; Moore, 2008). For both types of stimuli, the total bandwidth was from 50 to 6,805 Hz. More filters were used than in similar studies conducted in English or Chinese, because it turned out during a pilot study that words were not intelligible when fewer than 10 were used. Kong and Zeng (2006) note that, in order for noise-vocoded signals to be comparable to natural, unprocessed stimuli in terms of tone recognition, more than 30 channels are needed. Thus, both 10-band stimuli and 15-band stimuli still sounded far from natural speech. Passbands were 6 dB down at cutoff frequencies with the roll-off of a sine in the complex spectrum domain, as specified in Praat (Boersma and Weenink, 2019). The amplitude envelope from each frequency band was extracted using the algorithm implemented in Praat, in which intensity values were first squared and then convoluted with a Gaussian 64 ms analysis window (Kaiser-20), while removing pitch-synchronous oscillations above 50 Hz. Because the typical *f*_*o*_ range is above 50 Hz for both male and female voices, the low-pass filter with 50-Hz cutoff frequency ensured that the pitch information in the original speech would be removed (Loizou, 2006; Wilson et al., 2016). The envelopes obtained were used to modulate band-pass filtered noise that corresponded to the same frequency ranges as the source. The resulting stimuli preserved temporal information of the original speech while removing the spectral details within each band, including the *f*_*o*_ information. In practice, they sound more or less like a whispered speech. Sample spectrograms of the original speech and its noise-vocoded version are shown in Figure 3.

**Table 2:** Cutoff frequencies for the 10-band and 15-band stimuli.

**10-band stimuli**			**15-band stimuli**		
**Band #**	**Lower (Hz)**	**Upper (Hz)**	**Band #**	**Lower (Hz)**	**Upper (Hz)**
1	50	156	1	50	120
2	156	303	2	120	200
3	303	506	3	200	303
4	506	785	4	303	431
5	785	1,172	5	431	589
6	1,172	1,705	6	589	785
7	1,705	2,442	7	785	1,029
8	2,442	3,460	8	1,029	1,331
9	3,460	4,865	9	1,331	1,705
10	4,865	6,805	10	1,705	2,170
			11	2,170	2,746
			12	2,746	3,460
			13	3,460	4,345
			14	4,345	5,443
			15	5,443	6,805

### 2.3. Participants

Forty-four native speakers of Tokyo Japanese (18–22 years old, 18 males, 26 females), recruited at Keio University Hiyoshi Campus located in Yokohama City, participated in the study as listeners. All the participants grew up in Tokyo or certain areas of Kanagawa, Saitama, or Chiba, where Tokyo Japanese was spoken. While it would have been more desirable if both of the participants' parents were also Tokyo Japanese speakers, it was impossible to recruit enough participants with such a strict restriction. Many people who currently live in Tokyo originally come from an area outside the city. However, it was made sure that at least one of their parents was a native speaker of Tokyo Japanese. None of them reported any known history of a hearing or speaking disorder. The experiment lasted for roughly 1 h for each participant and the participants were paid for their participation. During data analysis, data from four participants were dropped because it turned out their data were not properly collected (e.g., a listener heard only the male voice stimuli due to the experimenter's mistake). Thus, the data from 40 listeners were examined in the analysis. The experiments were conducted based on approved ethics protocols for the treatment of human subjects.

### 2.4. Procedure

The experiment was conducted on the Hiyoshi Campus of Keio University. Listeners were tested individually in a quiet room. The stimuli were presented monaurally over circumaural Audio-Technica ATH-M50x headphones at a comfortable listening level. The stimuli were played using the SuperLab stimulus presentation software (Cedrus, 2009) installed on either a MacBook Air or a MacBook Pro. All responses from the listeners were logged using SuperLab.

The actual trials were preceded by two phases of practice sessions. The purpose of the first phase of practice sessions was to get used to the task required in the actual trials, and the purpose of the second phase was to familiarize listeners with noise-vocoded speech. In the first phase, listeners heard the original, naturally-produced speech, based on which noise-vocoded speech to be presented in the actual trials was created. Because test words used in the actual trials virtually exhausted all minimal pairs of words that would be suitable for this study, the same set of words as those used in the actual trials were used in practice sessions. At the beginning of each trial, one of the test words appeared on the computer screen. Then, the listener heard both the target word produced in a carrier phrase and its counterpart produced in the same carrier sentence in a randomized order. The carrier phrase used was *watashi wa* — *ga suki*, I TOPIC — NOM like, “I like —.” After listeners heard two sentences in which either the target word or its counterpart was embedded, the instruction appeared on the computer screen, asking them to press the key labeled “1” if they thought the word they saw corresponded to the word they heard in the first sentence, and press the key labeled “2” if they thought the word they saw corresponded to the word they heard in the second sentence (identification with two-alternative forced-choice). The test words were presented in the way indicated in “Orthography” in Table 1, which was most commonly written in Japanese. For Chinese characters (*kanji*) which could be read in more than one way, the superscript was written to indicate how they should be read. Listeners had to respond within 4 s after the instruction to press a key disappeared on the computer screen. At the end of each trial, feedback was given to the listeners. Because the purpose of the first phase of practice session was only to make listeners get used to the task required in the actual trials, there were only five trials which used five test words out of the ten as target words. The target words used for the first phase of practice sessions are shown in Table 3. The table also indicates the orders in which target words and their counterparts were presented. The order in which the five target words were displayed on the computer screen was randomized for each listener^1^.

**Table 3:** Test words used for the first phase of practice sessions and the order of auditory presentation within each pair.

**Pair**	**Visual presentation (target word)**	**Auditory presentation**
hana	Accented	Unaccented first
hasi	Accented	Accented first
hati	Unaccented	Unaccented first
osu	Unaccented	Accented first
sita	Accented	Unaccented first

The purpose of the second practice phase was to familiarize listeners with noise-vocoded speech. Studies have shown that a certain amount of exposure is often needed for noise-vocoded speech to become intelligible (Shannon et al., 1995; Fu et al., 1998; Davis et al., 2005). Considering the difficulty of the task required in the actual trials, the stimuli that listeners heard in the second phase of practice sessions was identical to those that they would hear in the actual trials. This phase of practice sessions consisted of ten trials in which all test words were presented on the computer screen as a target word once. Similar to the first phase, feedback was given to listeners whether they responded correctly or not. After these two phases of practice sessions, the actual trials started.

In the actual trials, each block consisted of twenty trials in which all the test words appeared twice as target words. For one trial, after a target word appeared on the screen, the noise-vocoded sentence in which the target word was embedded was presented to the listener first, followed by the one in which its counterpart was embedded. In the other trial, after the target word appeared on the screen, the noise-vocoded sentence in which the target word's counterpart was embedded was presented to the listener first, followed by the one in which the target word was embedded. In each block, the order in which the twenty trials were presented was randomized. Each listener went through ten blocks: five blocks with the male voice stimuli and five blocks with the female voice stimuli. The number of frequency bands was a between-listeners factor; for the 40 listeners whose data were analyzed, the same number of listeners (20 listeners) heard either ten blocks of 15-band stimuli or ten blocks of 10-band stimuli, resulting in 200 trials per listener (10 words × 2 repetitions × 5 blocks × 2 voices).^2^ Within each group of listeners, ten listeners heard the male voice first and the remaining ten heard the female voice first. No feedback was given in the actual trials.

### 2.5. Analysis

Although data were collected from 44 listeners, the data from four participants (two males) were dropped, as explained in Section 2.3. Thus, in the analysis, the data from 40 listeners (18 males) were included. Out of 40 listeners, ten heard the male voice first with 10-band stimuli, ten heard the female voice first with the 10-band stimuli, ten heard the male voice first with the 15-band stimuli, and ten heard the female voice first with the 10-band stimuli. Between listener factors were the number of frequency bands (10-band stimuli or 15-band stimuli) and the presentation order (male voice first or female voice first) while voice (male voice and female voice) was a within subject factor. In order to assess how well listeners distinguished final-accented words and unaccented words, the discriminability score *d'* was calculated while taking the listeners' bias (bias toward final-accented words or unaccented words) into account (Macmillan and Creelman, 2004). The calculated *d'* values were analyzed using one-sample *t*-tests against the null hypothesis that *d'* = 0, which would be the case when final-accented words and their unaccented counterparts are not discriminable. In order to examine the effects of voice (male or female voice) and the number of frequency bands on the discriminability of final-accented and unaccented words, a linear mixed-effects regression was applied to the data collected using the lme4 package (Bates et al., 2014) in R Core Team (2014) with *d'* values as the dependent measure. Order (whether the listeners heard female voice first or male voice first), Voice (whether the noise-vocoded speech was created from the male voice or female voice), the number of frequency bands of the stimuli (10-band stimuli or 15-band stimuli), and their interactions were coded as fixed effects. Listeners and test words were coded as random intercepts, and their random slopes were also included in the model.

### 2.6. Results

#### 2.6.1. Discriminability and Bias

**Figure 4:**
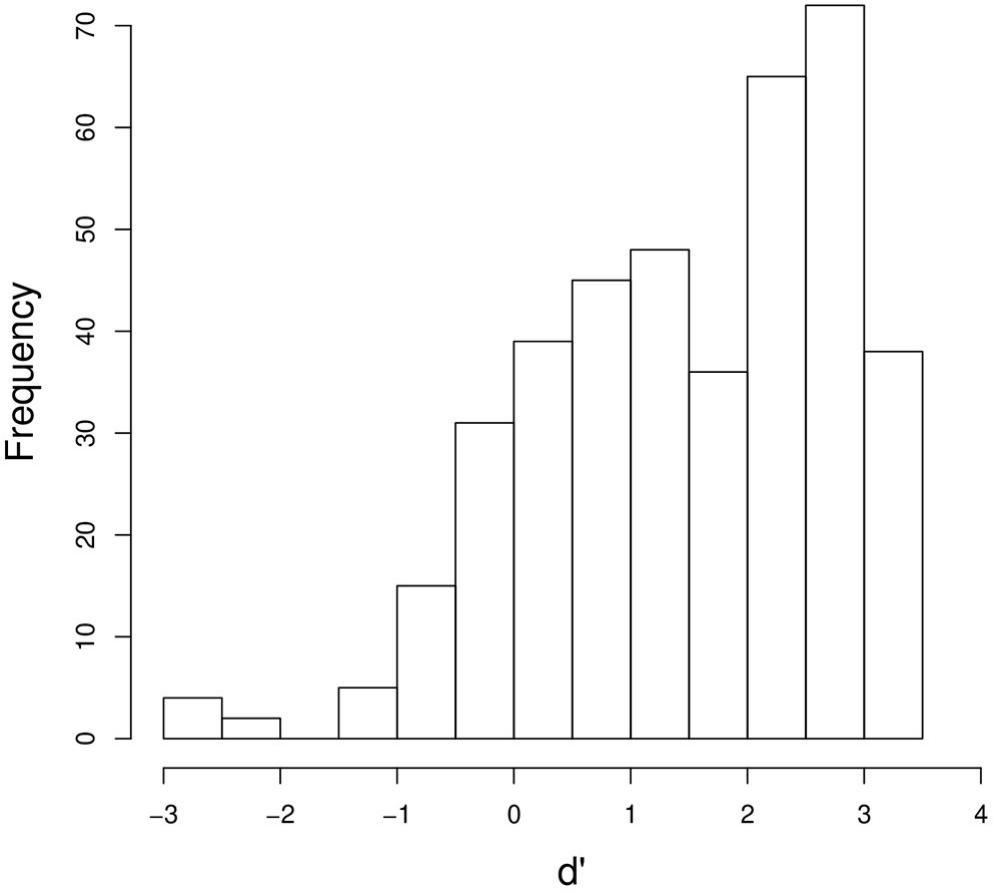
The distribution of the mean *d'* values for each listener for each word in each of the male or female voices.

In order to examine if final-accented words and unaccented words were distinguished at a rate better than chance, the mean *d'* values were calculated for each listener within each combination of word and speaker voice condition. In the analyses, responses to accented words when accented words were presented were treated as hits, and responses to accented words when unaccented words were presented were treated as false alarms. Responses to unaccented words when accented words were presented were treated as misses, and responses to unaccented words when unaccented words were presented were treated as correct rejections. Figure 4 shows the distribution of the mean *d'* values (n = 400; five pairs × two voices × 40 listeners). *T*-tests indicate that *d'* was significantly above zero (*t*(39) = 13.1, *p* < 0.0001) with a mean *d'* of 1.49 (SE = 0.11). The result indicates that, although the primary cue to pitch accent, *f*_*o*_, and its harmonics had been removed from the stimuli, listeners were able to distinguish final-accented words and unaccented words significantly better than chance. It suggests that some acoustic properties associated with pitch accent were present in the stimuli, enabling the listeners to identify the words they heard. This result provides converging support for Sugiyama (2017) that *f*_*o*_ and its harmonics are not the only cues to pitch accent. While the stimuli used in Sugiyama (2017) and the current study were created using different methods, they both lacked *f*_*o*_ and its harmonics, suggesting that the results found in both studies were not an artifact of some idiosyncratic properties contained in the stimuli, but some acoustic correlates remaining in the signal gave rise to the percept of pitch.

*c* scores were also calculated to assess if listeners' responses were biased toward final-accented words or unaccented words. In the analysis, positive *c* values would indicate listeners' bias toward unaccented words and negative values toward accented words. The mean was near zero (mean = 0.02, SE = 0.01). One-sample *t*-tests run against *c* = 0 were not reliable [*t*_(39)_ = 1.1, *p* > 0.1], indicating that the listeners were not biased toward identifying the stimuli as final-accented words or unaccented words. Past studies that used natural speech reported a listeners' tendency to respond that they heard accented words rather than unaccented words (Sugito, 1982; Vance, 1995) whereas, Sugiyama (2017) that used both natural speech and edited speech which had no *f*_*o*_ found the opposite trend. Considering the results from these past studies and the current study together, there seems to be no strong, consistent bias for listeners to identify words as final-accented words or unaccented words. It may be that listeners' responses depend on the exact nature of the stimuli used in each particular study. Because the main interest of the present study concerns listeners' discriminability of final-accented words and its unaccented counterparts, *d'* will be further examined below.

#### 2.6.2. Factors That Affect Word Discriminability

While *t*-tests show that the listeners were able to distinguish final-accented and unaccented words with above chance accuracy, they do not show how factors such as the number of frequency bands, the trial order (whether listeners heard the male voice first or the female voice), and the sex of the stimuli's voice affected the listeners' performance. In order to examine their effects on word identification, listeners' responses were fitted with linear mixed-effects regression models with the fixed effects of the number of frequency bands, Trial Order, and Voice, and the random effects of Listener and Pair. The maximum random effect structure justified by maximum likelihood test was: *d'* ~ the number of frequency bands * Trial Order * Voice + (1+Voice∣Listener) + (1+the number of frequency bands * Trial Order+Voice∣Pair). Because convergence was obtained using the maximal model, no simplification was made to the random effects structure.

**Figure 5:**
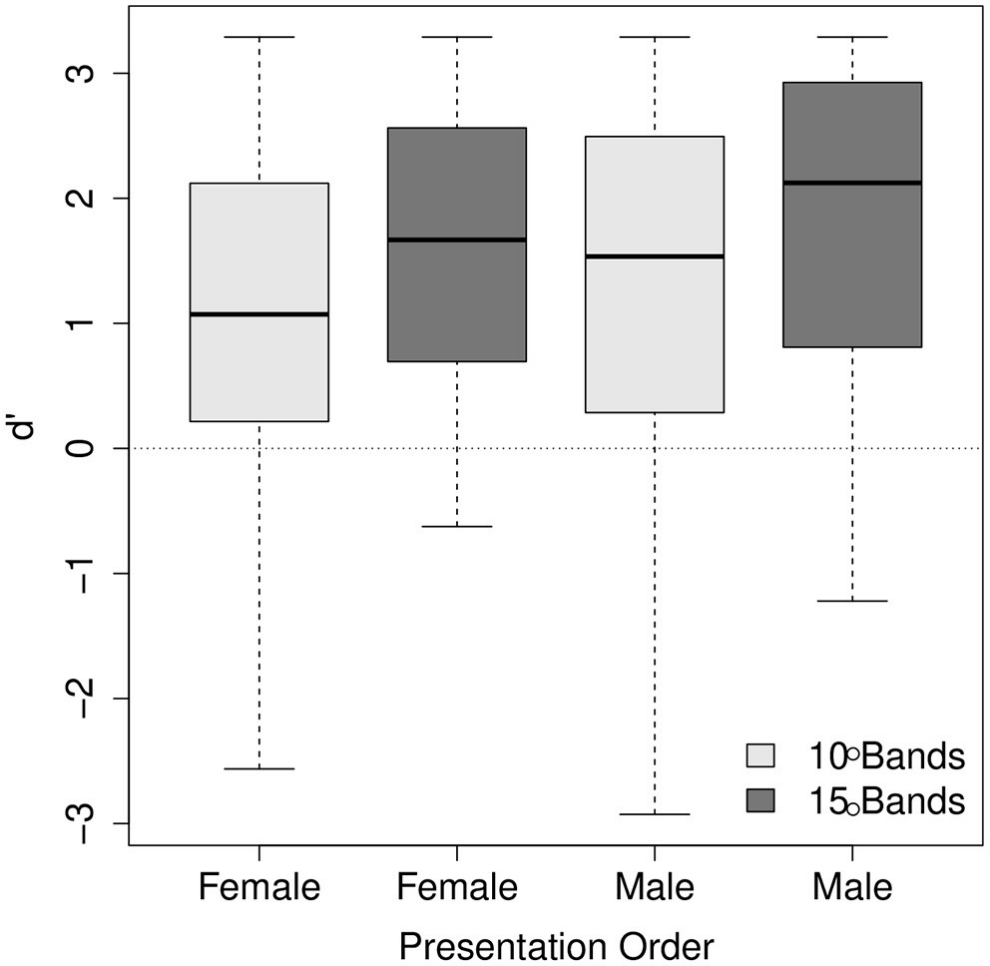
The interaction between the number of frequency bands and the order of presentation. In the x-axis, the labels “Female” and “Male” indicate the voice listeners heard first.

**Figure 6:**
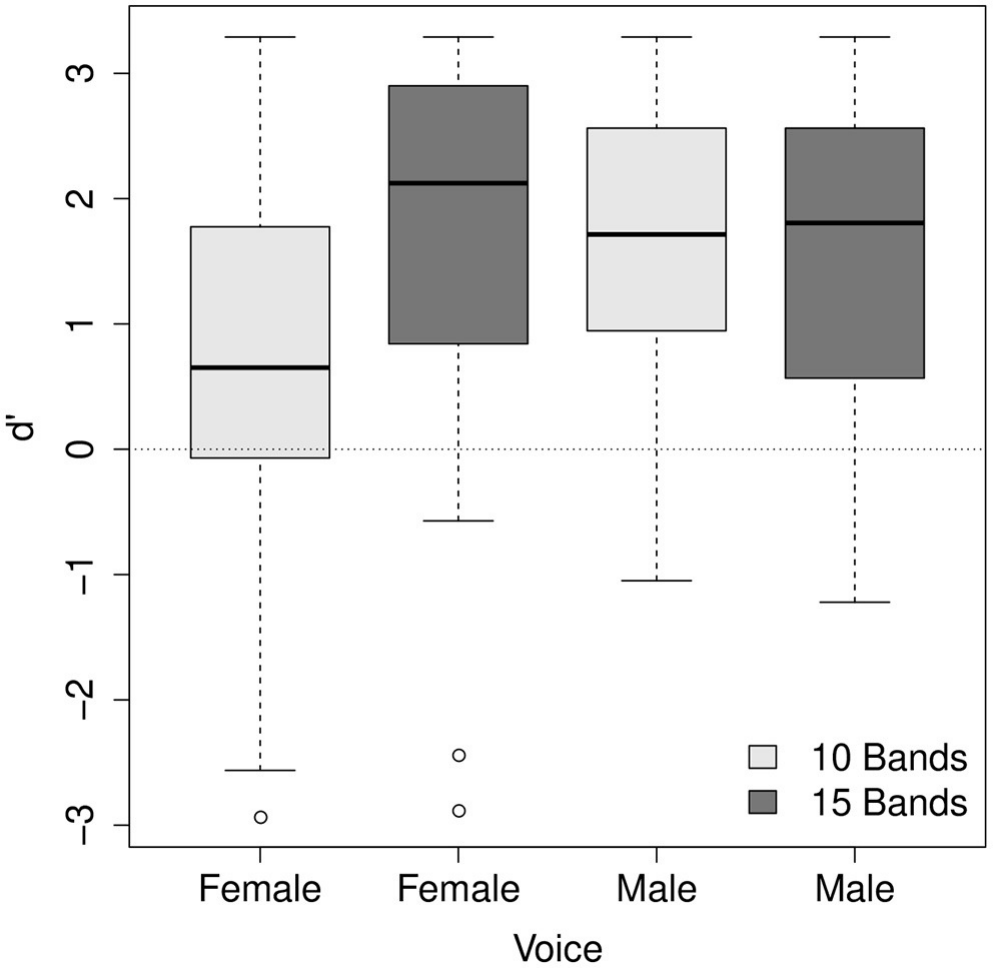
The interaction between the number of frequency bands and voice.

The main effects of the number of frequency bands and the stimuli's voice were significant (χ^2^ = 16.83, df = 4, *p* < 0.01; χ^2^ = 16.98, df = 4, *p* < 0.01, respectively). *d'* was higher for the stimuli with 15 frequency bands than for 10 frequency bands (mean = 1.74, SE = 0.09 for 15 frequency bands; mean = 1.23, SE = 0.09 for 10 frequency bands). This is consistent with the literature that speech intelligibility typically increases as the number of frequency bands increases (e.g. Shannon et al., 1995; Kong and Zeng, 2006). *d'* was higher for the male voice than the female voice (mean = 1.68, SE = 0.08 for the male voice; mean = 1.30, SE = 0.1 for the female voice), indicating that final-accented words and unaccented words were more discriminable for the male voice than for the female voice. The main effect of trial order was not reliable (χ^2^ = 3.65, df = 4, *p* > 0.1) while a significant two-way interaction between the number of frequency bands and trial order was found (χ^2^ = 14.91, df = 3, *p* < 0.01). The two-way interaction between the number of frequency bands and voice (χ^2^ = 15.15, df = 3, *p* < 0.01) was also significant. The interaction between trial order and voice was not significant (χ^2^ = 1.79, df = 3, *p* > 0.1). The three-way interaction was found significant (χ^2^ = 15.15, df = 4, *p* < 0.01). The nature of the interaction between the number of frequency bands and trial order is illustrated in Figure 5 and the interaction between the number of frequency bands and voice in Figure 6. As Figure 5 shows, for some reason, between the same number of frequency bands, *d'* scores were higher for the listeners who heard the male voice first than for those who heard the female voice first. However, the interaction between the number of frequency bands and the order of presentation does not seem so visually apparent. Figure 6 shows that the listeners' accuracy was higher for the 15 band stimuli than for the 10 band stimuli for the female voice, but the accuracy was more or less the same for the male voice. The results for the female voice are expected and consistent with the literature. It is somewhat unexpected and interesting why the listeners' accuracy was not affected by degrees of signal degradation for the male voice. The discrepancy between the male voice and female voice data will be explored in the following section.

#### 2.6.3. Word Analysis

As explained in Section 2.1, the test words used in the present study were all relatively familiar to Japanese speakers, reducing the likelihood that the listeners' performance would be affected by whether or not listeners were familiar with the words. Therefore, when some words tend to have better intelligibility than others within the same condition (the same number of bands in the same voice), it would be attributed to factors other than word familiarity, such as some acoustic information contained in the stimuli.

**Figure 7:**
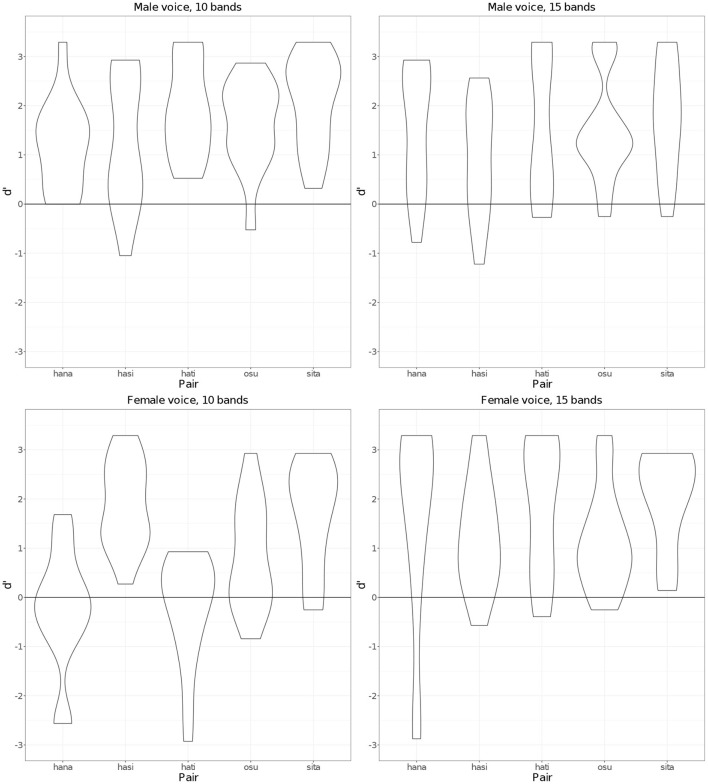
*d'* scores by pairs for 10 and 15 frequency bands in male and female voices.

Figure 7 shows *d'* scores for each pair of words associated with the 10- and 15-band conditions in male and female voices. As the figure shows, there seems to be no consistent, substantial effect of pairs on the listeners' word discriminability. Overall, discriminability was less affected by the number of bands for the male voice stimuli than for the female voice stimuli. The lowest mean *d'* score for the female voice's 10 band stimuli in Figure 6 seems to be related to the low *d'* scores for the two pairs of words, *hana* and *hati*. Considering that these words were concrete nouns and the resulting sentences seemed more meaningful than other words, it was rather unexpected that these pairs had lower discriminability than other pairs. That *hati* was less well perceived makes sense, since the second syllable in *hati* often undergoes devoicing; however, it is surprising that *hana* was also less well perceived. This needs to be researched further. Across four different conditions, which are represented as separate graphs in Figure 7, the *sita* pair has relatively high *d'* scores in all conditions. Even in normal speech, the first vowel /i/ is typically produced as a devoiced vowel because it occurs between voiceless consonants. This means that there was a smaller difference between what listeners usually hear in normal speech and what was presented in the current experiment than for the other pairs, which may have made it somewhat easier for listeners to distinguish the pair.

## 3. Experiment 2: Acoustic Study

The preceding perception study found that pairs of words that differ only in terms of accent were distinguishable at a rate better than chance even when *f*_*o*_ information had been removed, suggesting that some other acoustic properties served as cues for accent. This section aims to identify these acoustic cues. Acoustic properties typically measured in search of acoustic correlates of stress, tone, or pitch accent are duration, fundamental frequency, intensity, and formant frequency (Fry, 1958; Beckman, 1986; Everett, 1998; Gordon and Roettger, 2017). Because fundamental frequency had been removed in the stimuli used in the perception study, duration, intensity, and formant frequency were measured. Furthermore, for duration and intensity, in order to assess how their acoustic difference between final-accented and unaccented words would relate to the listeners' performance, correlation analyses were conducted.

### 3.1. Results

#### 3.1.1. Duration

**Figure 8:**
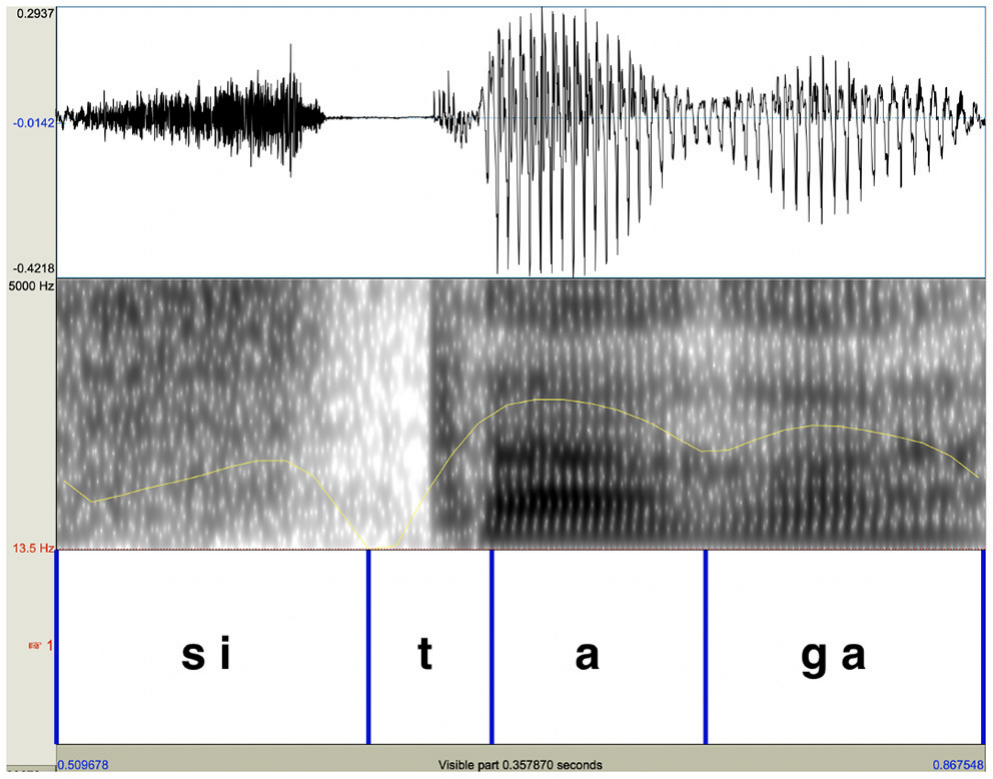
Segmental boundaries were placed using the original natural speech. The duration of the word-final vowel of the word /sitá / was calculated by dividing the vowel duration by the word-final syllable duration.

Past studies typically found no substantial effect of pitch accent on syllable duration or vowel duration in Japanese (Beckman, 1986; Kaiki et al., 1992; Cutler and Otake, 1999), while Itahashi (2005) notes that accented vowels are longer than unaccented vowels. In the present study, syllable duration and vowel duration were measured to assess whether these durational properties were affected by accent. In measuring duration, the original natural speech was used because segment boundaries were more reliably identifiable when voicing was present. For syllable duration, durations of the word-final accented syllables and those of the word-final unaccented syllables were compared. In comparing syllable duration of the two types of words, syllable durations measured were divided by the entire durations of the corresponding words. In comparing vowel duration, durations of the word-final vowel were divided by the durations of the entire syllables that contained the vowel. Vowels were identified as portions which had relatively clear formant structure in the spectrograms and evidence of voicing in waveforms, which was also confirmed by auditory inspection. Figure 8 shows an example of how boundaries were placed for the syllable /tá /. Because the closure for /ɡ/ was often incomplete due to lenition, its onset was difficult to identify from its waveform and spectrograms. Therefore, the intensity was referred to (the yellow line in the spectrogram) and the onset of the consonant was identified as the time when the intensity reached its minimum.

**Figure 9:**
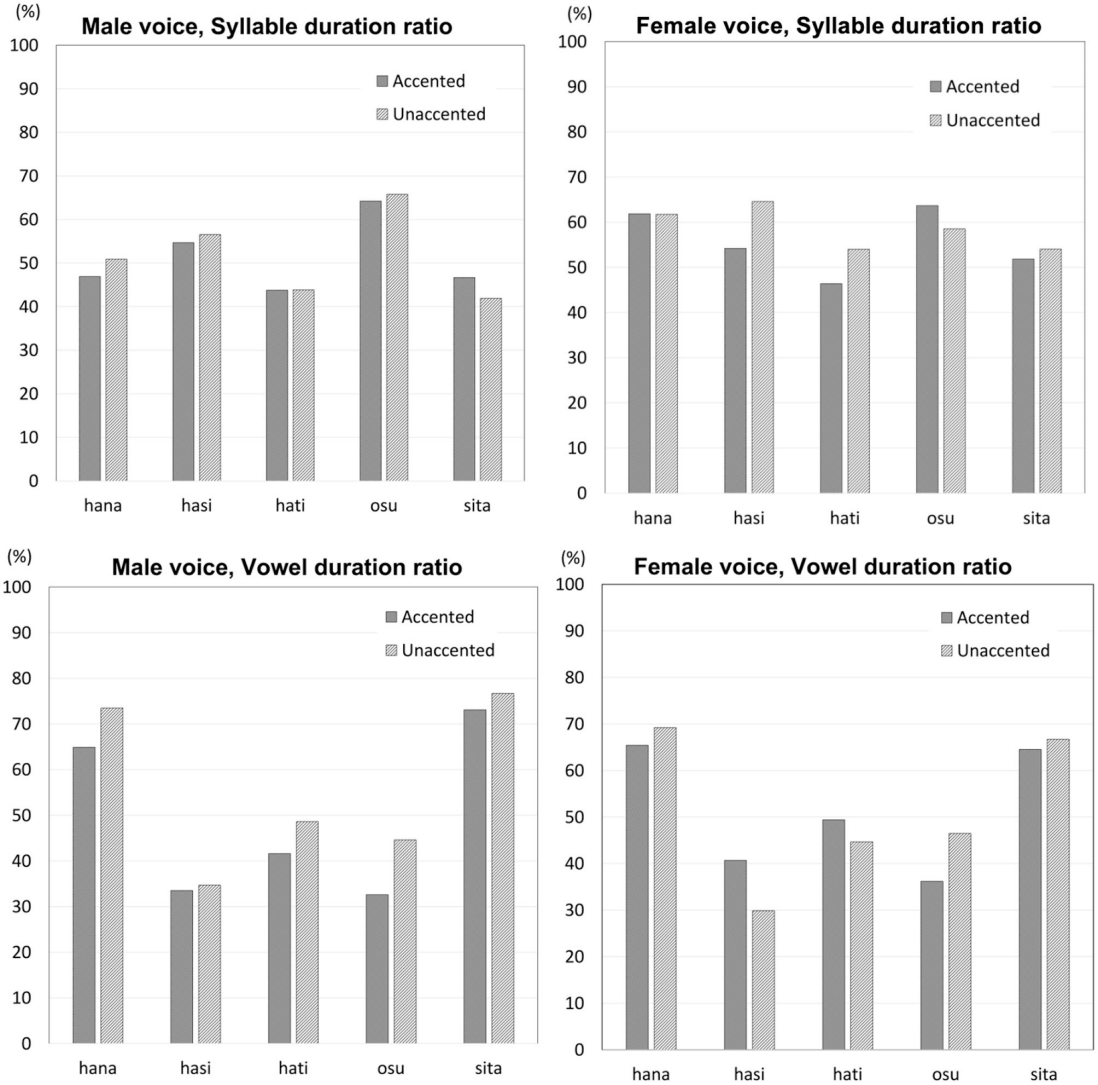
Syllable duration ratios (upper panels) and vowel duration ratios (lower panels) for the male voice stimuli and the female voice stimuli.

Syllable duration showed no regular or major difference between final-accented words and unaccented words for either male or female voice stimuli (Figure 9, upper panels). For the male voice stimuli, syllable duration was overall similar between the two types of words. For the female voice stimuli, syllable duration showed some difference for some of the words, but the difference showed no regular pattern. While syllable duration was clearly longer for unaccented words for /hasi/ and /hati/, the pattern was opposite for /osu/. Statistically, Wilcoxon signed rank exact test showed no effect of accent for the male voice (V = 5, *p* > 0.1) or the female voice (V = 4, *p* > 0.1).

Vowel duration showed different results for the male voice stimuli and the female voice stimuli (Figure 9, lower panels). For the male voice stimuli, vowel duration was longer for unaccented words than final-accented words. Itahashi (2005) proposes that vowel duration would be longer when accented, but the present study found the opposite pattern. Vowel duration occupied 56 percent of the syllable (ME = 8.4) when unaccented while it was 49 percent (ME = 8.3) when accented. For the female voice stimuli, vowel duration showed no consistent difference between final-accented and unaccented words. Vowel duration did not differ much between the two types of words for /hana/ and /sita/. Vowel duration was longer when accented than when unaccented by roughly 10 percent for /hasi/, but the pattern was opposite for /osu/. Wilcoxon signed rank exact test on vowel duration found that the difference between final-accented words and unaccented words was marginal for the male voice (V = 0, *p* = 0.06), but the difference was not significant for the female voice (V = 8, *p* > 0.1).

Although no clear consistent difference in duration was observed between final-accented and unaccented words, it was possible that listeners' judgments were affected by duration. That is, there may be more responses for final-accented words or unaccented words when syllable duration or vowel duration was longer. In order to investigate this possibility, a correlation analysis was conducted to examine if listeners' discriminability was correlated with duration. Spearman's rank correlation analysis found no significant correlation between syllable duration and the discriminability (*d'*) for the male voice stimuli or the female voice stimuli (ρ = 0.12, *p* > 0.1 and ρ = 0.03, *p* > 0.1, respectively). The results for vowel duration were similar to the ones for syllable duration^3^. Spearman's rank correlation analysis found no significant correlation between vowel duration and discriminability for the male voice stimuli or the female voice stimuli (ρ = –0.11, *p* > 0.1 and ρ = 0.01, *p* > 0.1, respectively).

#### 3.1.2. Intensity

Previous studies that analyzed natural speech are consistent in finding that intensity was greater for accented syllables than unaccented syllables (Weitzman, 1970; Beckman, 1986; Sugiyama, 2017). However, it was not certain if the difference was large or consistent enough to serve as a reliable cue to pitch accent. In the present study, intensity difference between the final-accented words and unaccented words was measured by subtracting the minimum intensity of the following particle from the maximum intensity of the second syllable (either accented or unaccented) of the test words. Then, intensity difference was compared between final-accented words and unaccented words. If intensity difference was greater for final-accented words than unaccented words, as previous studies had shown, intensity difference would exhibit positive values.

**Figure 10:**
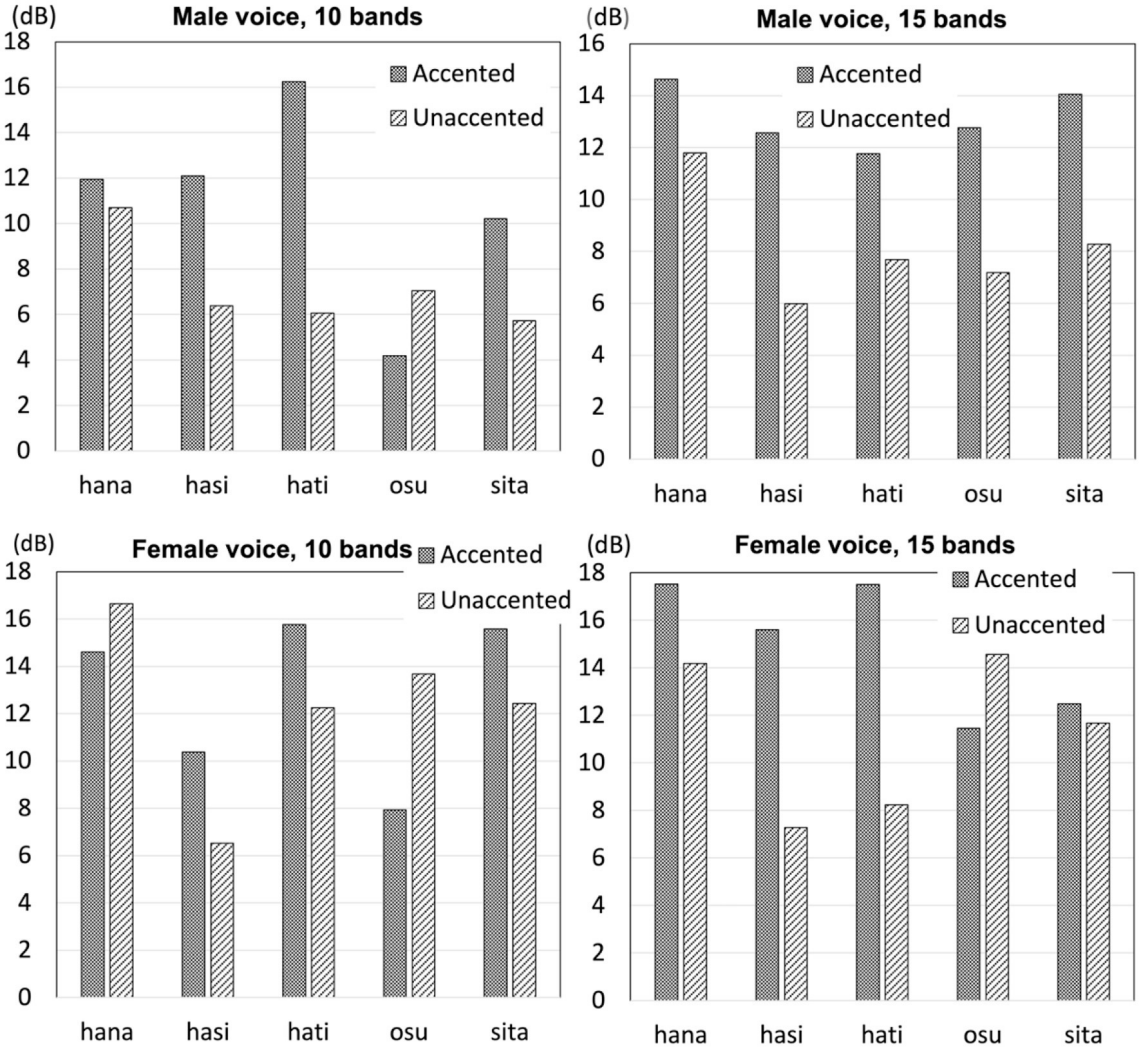
Intensity difference from the word-final syllable to the following particle for the 10 band stimuli and the 15 band stimuli for the male voice and female voice stimuli.

Overall, as illustrated in Figure 10, it appeared that intensity difference was greater for final-accented words than unaccented words. For the male voice 10 band stimuli, intensity difference was greater for all final-accented words than unaccented words but one pair. For the male voice 15 band stimuli, intensity difference appeared to be consistently and clearly greater for final-accented words than unaccented words. The results were less consistent for the female voice stimuli. For the 10-band stimuli, final-accented words had greater intensity difference than unaccented words for three pairs and the pattern was opposite for two pairs. For 15 band stimuli, intensity difference was clearly greater for final-accented words than unaccented words for three pairs and it was slightly greater for final-accented words than unaccented words for one pair. The pattern was opposite for one pair. According to Flanagan (1957), the difference limen for intensity is approximately ± 1 dB, or ±12 percent for synthetic vowels. In the current experiment, the stimuli presented to listeners probably contained more variabilities than ones used in Flanagan (1957); listeners heard the test words in a sentential context and the test words were created based on sentences produced naturally, as opposed to creating them completely synthetically. These factors are likely to make it more difficult for listeners to distinguish words in each pair, making the difference limen larger than ± 1 dB, or ±12 percent. As shown in Figure 10, intensity difference between final-accented and unaccented words was much larger than 1 dB for many pairs.

Wilcoxon signed rank exact test found intensity fall was not significant for the male voice stimuli (*V* = 13, *p* > 0.1) or the female voice stimuli (*V* = 9, *p* > 0.1) for the 10 band stimuli. For the 15 band stimuli, the difference was marginal for the male voice stimuli (*V* = 15, *p* = 0.06), but the difference was not significant for the female voice stimuli (*V* = 13, *p* > 0.1). Even though the intensity difference between the word-final syllable and the following particle appeared to be greater for final-accented words than unaccented words, statistical analyses found no significant difference between the two types of words.

In order to investigate if listeners' responses were affected by intensity, a correlation analysis was conducted to examine if their responses were correlated with their discriminability. The results of Spearman's rank correlation found no significant correlation with intensity for the male voice with 10 bands or 15 bands (ρ = 0.10, *p* > 0.1; ρ = –0.09, *p* > 0.1, respectively). Similarly, listeners' discriminability was not correlated with intensity for the female voice with 10 bands or 15 bands (ρ = 0.02, *p* > 0.1; ρ = 0.08, *p* > 0.1, respectively). The results did not show any evidence that listeners' responses were influenced by intensity even though the acoustic analysis showed a tendency for final-accented words to have a greater intensity difference than unaccented words.

#### 3.1.3. Formant Frequency

As hypothesized in the source-filter theory (Fant, 1960), the activity at the vocal folds (the source) and the shape of the oral cavity (the filter) are typically independent of each other. However, results from experimental studies in the past have shown that the reality is much more complex, with some studies finding a correlation between formant frequencies and the *f*_*o*_ while others finding no such correlation. Studies which examined whispered speech tend to find a positive correlation between intended pitch and formant frequencies (Meyer-Eppler, 1957; Thomas, 1969; Higashikawa et al., 1996; Heeren, 2015). On the other hand, studies that analyzed formant frequencies in speech produced naturally do not find such a correlation (Zee, 1980; Sugiyama and Moriyama, 2013; Kawahara et al., 2014). Furthermore, there is a debate over whether or not non-*f*_*o*_ acoustic properties are exaggerated in whispered speech (Liu and Samuel, 2004; Chang and Yao, 2007). While Liu and Samuel (2004) found that duration differences among different tones were exaggerated in whispered speech in Mandarin Chinese, Chang and Yao (2007) found no such tendency. Since no conclusive data exist as to the relation between *f*_*o*_ and formant frequencies, the first and second formant frequencies of the stimuli used in the perception experiment were measured.

**Figure 11:**
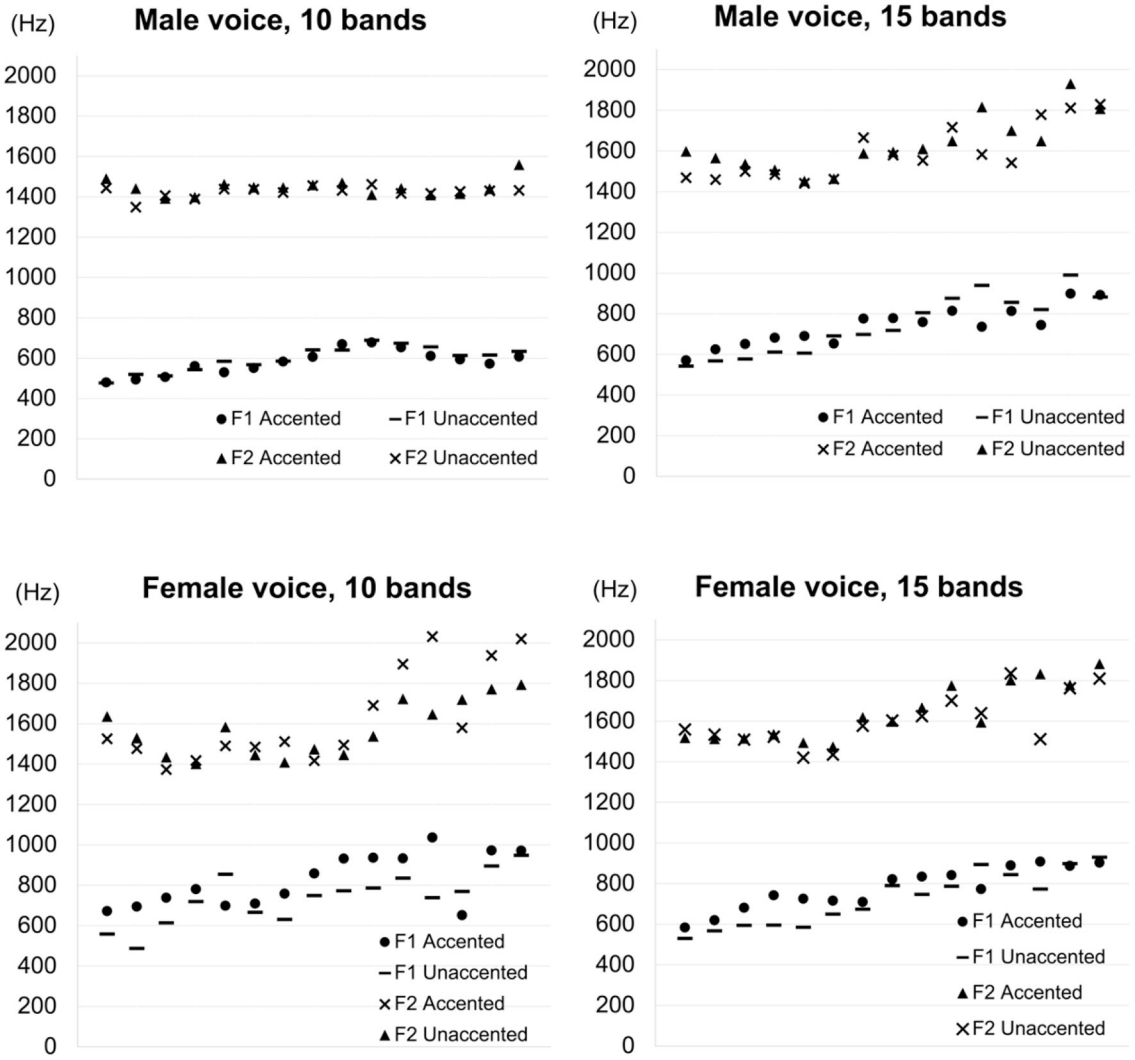
First formant frequencies (F1) and second formant frequencies (F2) of the particle for the 10 band stimuli and the 15 band stimuli for the male and female voices, normalized by the entire duration of the particle. In the legend, “Accented” indicates frequency values following final-accented words. “Unaccented” indicates frequency values following unaccented words.

In the present study, test words were produced in the middle of the carrier sentence (Section 2.4) and the whole sentence was presented to listeners. In natural speech, the most prominent difference between final-accented words and unaccented words appears in the *f*_*o*_ contour from word-final syllables into the following particle (which was *ga* in the present study). Thus, if secondary cues to pitch accent exist, it is reasonable to expect that the difference between the accented and unaccented types of words would also appear most clearly in that position. Based on this line of reasoning, the first two formant frequencies of the vowel in *ga* were measured. The upper panels in Figure 11 show the first and second formant frequencies of the particle for the male voices, with the left panel showing formant tracks for the 10 band stimuli and the right panel showing formant tracks for the 15 band stimuli. The formants were taken from the entire duration of the particle and normalized by the duration. Similarly, the lower panels in Figure 11 show the first and second formant frequencies of the particle for the female voices, with the left panel showing formant tracks for the 10 band stimuli and the right panel showing formant tracks for the 15 band stimuli. As the figure shows, it is hard to observe clear differences in formant frequencies between final-accented words and unaccented words in any of the four conditions. For the 10 band stimuli in the male voice, formant transitions are virtually identical for final-accented and unaccented words. For the rest of the three conditions, first formant frequencies seem a little higher when the particle followed the final-accented words than the unaccented words, especially earlier in the transitions. However, given that the *f*_*o*_ differences typically become greater toward the end of the particle, it is not clear if the frequencies differences in the first formant, if any, are related to *f*_*o*_ or accent information. Since the number of stimuli analyzed was limited in the present study, production studies with more data are needed to say anything conclusive about how accent affects formant frequencies.

## 4. Discussion and Conclusions

The present study was conducted to examine whether secondary cues exist in lexical pitch accent in Tokyo Japanese. In many languages, prosodic information is known to be realized in multiple acoustic properties. This has been widely attested in languages with lexical stress such as English (Fry, 1955, 1958), Dutch (Sluijter et al., 1997), Russian (Chrabaszcz et al., 2014), and Spanish (Ortega-Llebaria and Prieto, 2011). While the languages examined are relatively limited for lexical tone, extensive research in Chinese (mainly in Mandarin Chinese) has shown that tone is similar to stress in that it manifests itself in different acoustic dimensions, such as *f*_*o*_, amplitude contour and duration (Whalen and Xu, 1992; Fu et al., 1998; Liu and Samuel, 2004). Furthermore, in languages in which different types of phonation are utilized, certain tones occur with creaky voice or breathy voice to enhance the percept of those tones (Gordon and Ladefoged, 2001; Kuang, 2013; Brunelle and Kirby, 2016). Since such prosodic redundancy is less studied for Japanese, the present study aimed to assess if acoustic correlates exist in lexical pitch accent in Japanese. In short, the results obtained in the present study offer converging support for those found in Sugiyama (2017), showing that prosodic information is encoded in multiple acoustic dimensions in Japanese. Given that the stimuli were created using different coding methods in these two studies, it is unlikely that the listeners' judgements were based on some accidental, idiosyncratic acoustic signals left in the stimuli due to the particular method used to create the stimuli. Rather, secondary cues to Japanese pitch accent exist, which listeners make use of in word identification when the primary cue is not available.

The perception experiment showed that minimal pairs of final-accented words and unaccented words can be reliably identified when *f*_*o*_ and its harmonics were absent. The degrees of spectral degradation were varied by dividing the original signal into 10 and 15 frequency bands, but its effect was not clearly observed. At least two possible reasons, which are not mutually exclusive, can be speculated. One reason is that even 15 frequency band stimuli which theoretically preserved more of the acoustic information contained in the original speech than 10 frequency band stimuli were hard enough for listeners to distinguish final-accented words and unaccented words. A casual analysis of the listeners' responses during the first phase of the practice sessions suggested that Tokyo Japanese speakers could distinguish final-accented words and unaccented words almost perfectly in natural speech. Sugiyama (2017) also found that the listeners' performance was close to 100 percent when natural speech was used. In comparison, listeners' accuracy was much poorer even for the 15 band stimuli. If higher discriminability had been achieved for the 15 band stimuli, a clearer difference may have been observed between the two types of stimuli. The other reason is that 10 frequency band stimuli and 15 frequency band stimuli were perceptually similar, which can be observed in the more or less similar *d'* scores in all conditions. Typical studies of noise-vocoded speech use much broader filters, dividing the original speech into four bands or so. However, it turned out during pilot studies that it was impossible to distinguish final-accented words and unaccented words when the original speech was divided into less than 10 bands. In order for noise vocoded speech to be different enough from natural speech in terms of perceiving pitch accent, a pilot test suggested that noise vocoded speech should not be divided into more than 15 frequency bands. On the other hand, the effect of degradation might have been more clearly observed had the stimuli been divided into a greater number of bands, say, 20 frequency bands, instead of 15.

While the perceptual data clearly show secondary cues exist, acoustic measurements proved difficult to clearly identify them. Consistent with most studies, lexical pitch accent was not correlated with syllable duration (Beckman, 1986; Kaiki et al., 1992; Cutler and Otake, 1999). Lexical pitch accent showed no consistent effect on vowel duration for the female voice stimuli, but vowel duration was consistently longer for unaccented words than accented words for the male voice stimuli. However, correlation analyses between listeners' discriminability and syllable duration or vowel duration found no strong correlation between them. When the intensity difference between the word-final syllable and the following particle was measured for 10 band stimuli and 15 band stimuli for both male and female voices, the male voice's 15 band stimuli showed that the intensity difference was consistently and clearly greater for final-accented words than unaccented words. However, similar to duration, correlation analyses between listeners' discriminability and intensity difference did not show any strong correlation. The measurement of formant frequencies found that first formant frequencies were higher after accented syllables than unaccented syllables for both male and female voices. It may be that duration, intensity, and formant frequency interact and as a result, affect the perception of Japanese pitch accent. It is also possible that there were some cumulative effects from the verb following the noun phrase (test word and the particle). Japanese is known to have downstep, which is a lowering of the peak *f*_*o*_ and compression of the *f*_*o*_ range when a word follows an accented phrase (Pierrehumbert and Beckman, 1988). The results found in Sugiyama et al. (2021) suggest that, in normal speech, listeners make use of presence or absence of downstep in judging if the preceding word was accented or not. Although the effect would be weaker in noise-vocoded speech, it is not possible to completely eliminate the possibility that listeners used the acoustic information about downstep or its absence in identifying if the test words they heard were accented or unaccented. For future research, it would be interesting to investigate how listeners adapt to variabilities in speech and integrate acoustic information in perceiving lexical accent.

Research that compared the intelligibility of noise-vocoded speech in various languages indicates that segments can be identified with high accuracy (roughly 70 percent or more) even when the original speech was divided into as few as four bands (Shannon et al., 1995; Fu et al., 1998; Souza and Rosen, 2009; Ellermeier et al., 2015; Kishida et al., 2016). When it comes to prosodic information at the word level, stress, tone and pitch accent seem to vary in terms of how much they tolerate spectral degradation, suggesting that the degree of spectral redundancy and the way in which prosodic information is encoded are different depending on the prosodic type. In English, the lexical judgement of nouns and verbs that contrast only in the position of the primary stress (e.g., *object* and *permit*) is affected by duration, intensity, and *f*_*o*_ contour, showing that they all act as a cue to stress. Vowel quality is also known to co-vary with stress (Lehiste, 1970). These cues are so closely interlaced that it is difficult to determine which cue is the strongest for lexical stress in English (Peterson and Lehiste, 1960; Sluijter and van Heuven, 1996). For both Chinese tone and Japanese pitch accent, while *f*_*o*_ is the dominant cue, secondary cues seem much more salient for lexical tone in Chinese than for lexical pitch accent in Japanese. Tones can be identified with 65 percent accuracy with the amplitude contour alone (Kong and Zeng, 2006). As the present perception experiment showed, the discriminability of accent type is much poorer in the absence of *f*_*o*_. While it is the primary cue for both Chinese tone and Japanese pitch accent, the results of the present study suggest that the reliance on the primary cue is much stronger for Japanese pitch accent. The subsequent acoustic measurement found that syllable duration did not differ as a function of accent, although vowel duration was longer for unaccented words than accented words for the male voice stimuli. While the intensity difference was clearly and consistently greater for final-accented words than unaccented words for the male 15 band stimuli, considering that listeners' performance was more or less similar for the male 10 band and 15 band stimuli and the female 15 band stimuli, it is not certain if intensity alone would be sufficient to cue pitch accent. Furthermore, correlation analyses between these acoustic measures and the listeners' word discriminability showed no strong correlations. The formant analysis found that the first formant frequency was higher after accented syllables than unaccented syllables. Further corroboration is needed to determine if the first formant frequency alone can serve as a cue to pitch accent. Although reliable, the word discriminability of minimal pairs between final-accented words and unaccented words was relatively low in noise-vocoded speech, which suggests a lack of consistent, robust secondary cues to lexical pitch accent in Japanese. For future research, it would also be a good question to ask whether findings from the current study apply to other so called pitch accent languages. Swedish, Serbo-Croatian and Korean, for example, are said to have pitch accent (Gårding, 1977; Inkelas and Zec, 1988; Jun et al., 2006). It seems that pitch accent in these languages seems to have different phonetic properties and functions. It would be interesting to examine if findings from the current study in Japanese apply to other languages which have pitch accent.

The current study was limited in a number of ways. First, it used only five minimal pairs between final-accented words and unaccented words. For future research, it would be interesting to compare more minimal pairs with different accent types. Including words with initial accent or word-medial accent will be of particular interest because their pitch patterns are quite different from final-accented words and unaccented words. In addition, the current study was limited in that it used speech from only one female speaker and one male speaker, making it difficult to assess if differences found between the two speakers were gender differences or simply individual differences. Furthermore, since only one token was used for each word, acoustic analyses were compromised in their generality. Acoustic analyses which involve more repetitions of more words are necessary to determine what acoustic properties are present to serve as reliable secondary cues to lexical accent in Japanese.

## Data Availability Statement

The raw data supporting the conclusions of this article will be made available by the author, without undue reservation.

## Ethics Statement

The studies involving human participants were reviewed and approved by the Institute of Cultural and Linguistic Studies, Keio University. The patients/participants provided their written informed consent to participate in this study.

## Author Contributions

The author confirms being the sole contributor of this work and has approved it for publication.

## Funding

This research was supported by the Japan Society for the Promotion of Science (JSPS) Grant-in-Aid for Scientific Research (C) No. 19K00584.

## Conflict of Interest

The author declares that the research was conducted in the absence of any commercial or financial relationships that could be construed as a potential conflict of interest.

## Publisher's Note

All claims expressed in this article are solely those of the authors and do not necessarily represent those of their affiliated organizations, or those of the publisher, the editors and the reviewers. Any product that may be evaluated in this article, or claim that may be made by its manufacturer, is not guaranteed or endorsed by the publisher.

## Footnotes

^1^The listeners' responses in the first practice phase were also utilized to evaluate if their phonological representation of the accent pattern was the same as the accent pattern indicated in Lexical Properties of Japanese (Amano and Kondo, 1999). Even though a relatively stringent criterion was used in recruiting participants, variation among Tokyo Japanese speakers is well-known (Sugito, 1982; Vance, 1995; Nielsen, 2015). It turned out that three out of 40 listeners performed very differently during their first practice phase; their responses consisted of only incorrect responses and no responses. Indeed, their performance was actually better for the actual trials than for the first practice phase which used natural speech. Therefore, their responses were excluded. For 185 responses (5 words × 37 listeners), the accuracy was over 89 percent (165/185 correct responses). For the responses that were not correct (20 responses), 17 actually had no responses, all of which were the first word presented to them. This suggests that they were not used to the task and were not sure what to do. Given that only five out of ten words (one word from each pair) were used and the responses were from the practice session, this result provides only a rough measure, but it strongly suggests that the listeners' accent pattern was consistent with the one indicated in the database at least for the words presented during the first practice phrase.

^2^The design of the experiment was another reason for why test words were limited to five pairs. In order to make sure that each listener would complete the experiment within a hour or so, it was difficult to include more words.

^3^Segment boundaries are much less clear than syllable boundaries in noise-vocoded speech. Therefore, one might wonder why the correlation between vowel duration and discriminability was examined when the correlation between syllable boundaries and discriminability was not significant. Given how little was known about secondary cues to Japanese pitch accent, the analysis was conducted without making any assumptions.
